# Development of a physical geometric phantom for deformable image registration credentialing of radiotherapy centers for a clinical trial

**DOI:** 10.1002/acm2.13319

**Published:** 2021-06-22

**Authors:** Noriyuki Kadoya, Siwaporn Sakulsingharoj, Tomas Kron, Adam Yao, Nicholas Hardcastle, Alanah Bergman, Hiroyuki Okamoto, Nobutaka Mukumoto, Yujiro Nakajima, Keiichi Jingu, Mitsuhiro Nakamura

**Affiliations:** ^1^ Department of Radiation Oncology Tohoku University Graduate School of Medicine Sendai Japan; ^2^ Division of Radiation Oncology Faculty of Medicine Ramathibodi Hospital Mahidol University Bangkok Thailand; ^3^ Physical Sciences Peter MacCallum Cancer Centre Melbourne Vic. Australia; ^4^ Centre for Medical Radiation Physics University of Wollongong Wollongong NSW Australia; ^5^ Department of Medical Physics BC Cancer Agency Vancouver BC Canada; ^6^ Department of Medical Physics National Cancer Center Hospital Tokyo Japan; ^7^ Department of Radiation Oncology and Image‐Applied Therapy Kyoto University Kyoto Japan; ^8^ Department of Radiotherapy Tokyo Metropolitan Cancer and Infectious Diseases Center Komagome Hospital Tokyo Japan; ^9^ Department of Information Technology and Medical Engineering Human Health Sciences Graduate School of Medicine Kyoto University Kyoto Japan

**Keywords:** adaptive radiotherapy, credentialing, deformable image registration, physical phantom, quality assurance

## Abstract

**Purpose:**

This study aimed to develop a physical geometric phantom for the deformable image registration (DIR) credentialing of radiotherapy centers for a clinical trial and tested the feasibility of the proposed phantom at multiple domestic and international institutions.

**Methods and materials:**

The phantom reproduced tumor shrinkage, rectum shape change, and body shrinkage using several physical phantoms with custom inserts. We tested the feasibility of the proposed phantom using 5 DIR patterns at 17 domestic and 2 international institutions (21 datasets). Eight institutions used the MIM software (MIM Software Inc, Cleveland, OH); seven used Velocity (Varian Medical Systems, Palo Alto, CA), and six used RayStation (RaySearch Laboratories, Stockholm, Sweden). The DIR accuracy was evaluated using the Dice similarity coefficient (DSC) and Hausdorff distance (HD).

**Results:**

The mean and one standard deviation (SD) values (range) of DSC were 0.909 ± 0.088 (0.434–0.984) and 0.909 ± 0.048 (0.726–0.972) for tumor and rectum proxies, respectively. The mean and one SD values (range) of the HD value were 5.02 ± 3.32 (1.53–20.35) and 5.79 ± 3.47 (1.22–21.48) (mm) for the tumor and rectum proxies, respectively. In three patterns evaluating the DIR accuracy within the entire phantom, 61.9% of the data had more than a DSC of 0.8 in both tumor and rectum proxies. In two patterns evaluating the DIR accuracy by focusing on tumor and rectum proxies, all data had more than a DSC of 0.8 in both tumor and rectum proxies.

**Conclusions:**

The wide range of DIR performance highlights the importance of optimizing the DIR process. Thus, the proposed method has considerable potential as an evaluation tool for DIR credentialing and quality assurance.

## 1 INTRODUCTION

Worldwide, audit systems have been established for credentialing institutions for participant clinical trials. Clinically unacceptable differences in doses and image guidance potentially influence clinical outcomes.^1^, ^2^ Thus, dose delivery and image guidance are now evaluated by credentialing institutions for various clinical trials.^3^, ^4^, ^5^

Recently, deformable image registration (DIR) has become essential in radiotherapy, including multimodality image fusion and dose accumulation.^6^, ^7^, ^8^ Phase I clinical trials in which a treatment is adapted every ten fractions use the DIR‐based dose accumulation for evaluating treatment outcomes as well as reporting.^7^ Several studies have shown that the DIR accuracy greatly depends on both the DIR software and procedure (e.g., DIR parameter settings).^9^, ^10^, ^11^ Thus, DIR credentialing is necessary to achieve more reliable clinical trial results.

Investigators have validated the DIR accuracy using several digital phantoms provided by the American Association of Physicists in Medicine (AAPM).^12^, ^13^ The digital phantom‐based method evaluates the DIR software itself; however, an end‐to‐end quality assurance (QA) test is required, which ensures accurate data representation, image transfer, and integrity verification between image acquisition devices, image registration systems, and other radiotherapy systems that use image registration results.^12^, ^14^, ^15^ Physical phantoms allow institutions to conduct end‐to‐end QA tests. Wognum et al. used a porcine bladder phantom with fiducial markers to evaluate the DIR accuracy.^16^ This method can reproduce the actual anatomical deformation; however, this phantom was not an easy tool to handle, particularly for large‐scale clinical trials. Singhrao et al. developed a three‐dimensional head and neck physical phantom.^17^ These phantoms could mimic the Hounsfield unit (HU) values of actual anatomy and deformation. However, because these phantoms need special knowledge and materials, they are not suitable for DIR credentialing. Kirby et al. used a two‐dimensional deformable phantom with a single plane of the anatomy for a head and neck regions.^18^ Although this phantom could reproduce the tumor deformation using the inflated and deflated catheter, it could not reproduce the deformation patterns.^19^, ^20^ Moreover, this phantom had great potential for DIR credentialing; however, it could not simulate complicated anatomical changes owing to its simplicity. To the best of our knowledge, no studies have been conducted on DIR credentialing (i.e., an end‐to‐end test for DIR performance) for any clinical trial. To perform DIR credentialing, the development of a DIR physical geometric phantom suitable for DIR credentialing is required.

In this study, we developed a physical geometric phantom for the DIR credentialing of radiotherapy centers for a clinical trial and tested the feasibility of implementing the proposed phantom at multiple domestic and international institutions.

## 2 MATERIALS AND METHODS

### 2.1 Development of the physical phantom

**Fig. 1:**
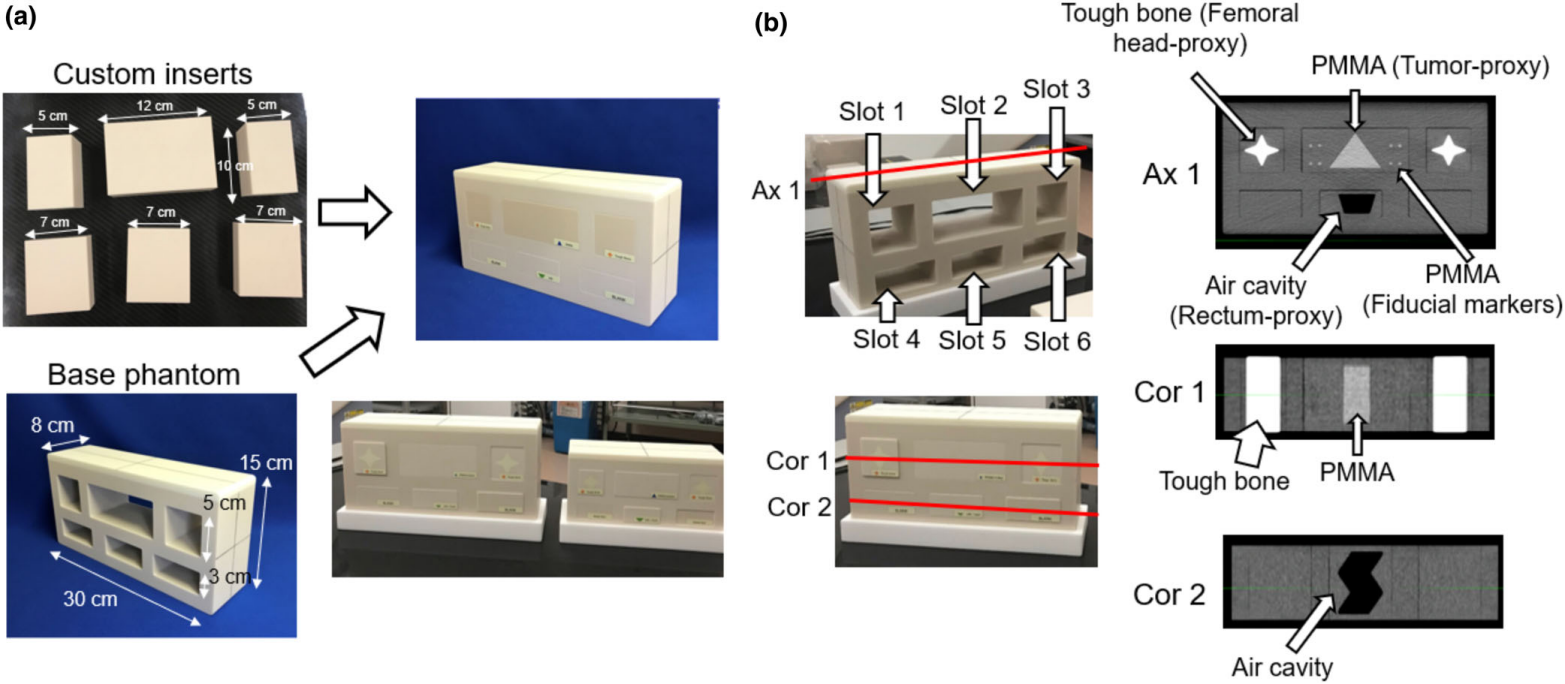
Overview of the proposed phantom. Figure (a) shows two base phantoms (large and small) and custom inserts. Figure (b) shows three representative CT images of the phantom [one axial image (Ax 1) and two coronal images (Cor 1 and Cor 2)]. The red lines in the phantom indicate the slice locations of three slices.

**Fig. 2:**
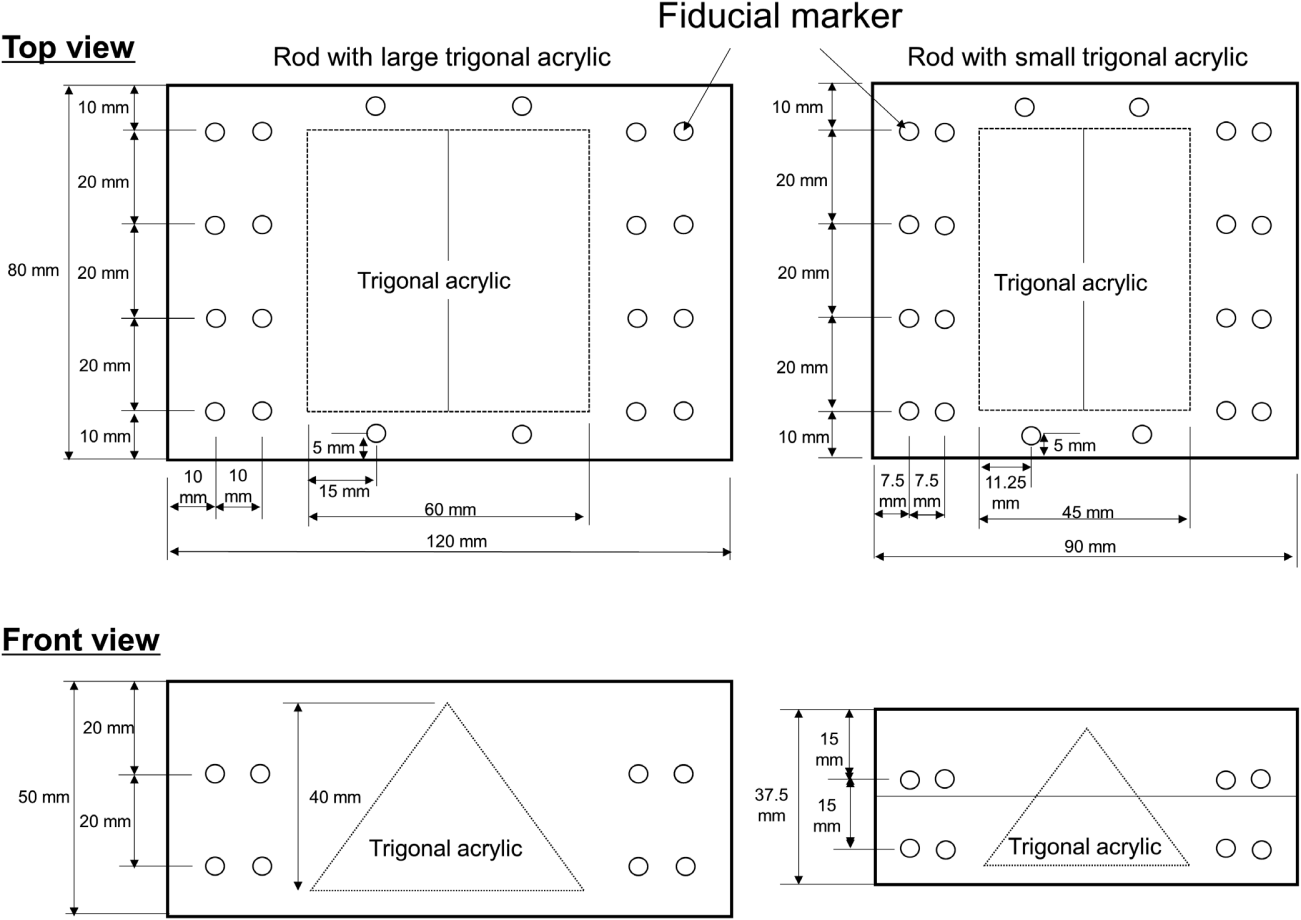
Layout of fiducial markers in inserts with a large trigonal acrylic and insert with a small trigonal acrylic. Circles indicate the marker's position.

The proposed DIR phantom comprised a base phantom and six custom inserts [Fig. 1(a)]. The base phantom was composed of a “tough water phantom” material (WD, Kyoto Kagaku Co. Ltd., Kyoto, Japan) and had six holes (slots 1–6). The density and effective atomic number of tough water were 1.017 g/cm^3^ and 7.42, respectively. The base phantom was designed to simulate various clinical situations using multiple custom inserts (e.g., tumor shrinkage and rectal filling). These custom inserts contained an internal object with different shapes and materials. This phantom is not an anthropomorphic phantom; the objective is to ensure that the phantom resembles the actual patient to the maximum possible extent without losing its geometric simplicity for quantitative analysis. Phantoms should be as small and light as possible to facilitate delivery. Therefore, the thickness of the entire phantom was set to 10 cm to reduce the phantom weight as much as possible while facilitating DIR evaluation. Herein, we selected six custom inserts that reproduce the features of the pelvic region in patients [Fig. 1(b)]. For slots 1 and 3, we inserted the inserts containing an octagonal “tough bone” (BE‐H, Kyoto Kagaku Co. Ltd) to simulate the right and left femoral heads. The octagonal “tough bone” had a length, width, and height of 8.0, 4.0, and 3.5 cm, respectively. The density and effective atomic number of the tough bone were 1.50 g/cm^3^ and 11.70, respectively. In actual patients, the shape of the femoral head is closer to a sphere than an octagon. The octagonal shape was adopted to facilitate a more detailed evaluation of the DIR accuracy using a shape with corners. For slot 2, we inserted two inserts with different sizes to simulate tumor shrinkage, namely, large and small trigonal polymethyl methacrylate (PMMA). The large trigonal PMMA had a length, width, and height of 6.0, 6.0, and 4.0 cm, respectively. The small trigonal PMMA had a length, width, and height of 6.0, 4.5, and 3.0 cm, respectively. The density of PMMA was 1.190 g/cm^3^. Moreover, these inserts had 40 fiducial markers (PMMA, 4‐mm Φ). These fiducial markers were evenly placed outside the trigonal PMMA. The detailed position of the fiducial marker is shown in Fig. 2. In actual patients, the shape of the tumor is closer to a sphere than a trigonal shape. The trigonal shape was adopted to facilitate a more detailed evaluation of the DIR accuracy using a shape with corners as well as femoral heads. For slot 5, we inserted three different custom inserts with different shapes simulating rectum deformation, namely, S‐shaped, inverted S‐shaped, and trapezoidal air cavities. These inserts had a length, base width, top width, and height of 6.0, 3.0, 4.0, and 2.0 cm, respectively. For slots 4 and 6, blank inserts (i.e., tough water inserts) were inserted. Moreover, a three‐quarter‐scaled base phantom was used to simulate the overall body shrinkage. This phantom was deigned to simulate weight loss; however, owing to technical issues with the production, everything, including the inserts, was scaled down to 3/4. To increase sensitivity for evaluating technical skills for DIR, we fabricated the proposed phantom with more deformation within the phantom, although this situation is unlikely to occur in actual clinical practices.

### 2.2 DIR at various phantom settings

**Fig. 3:**
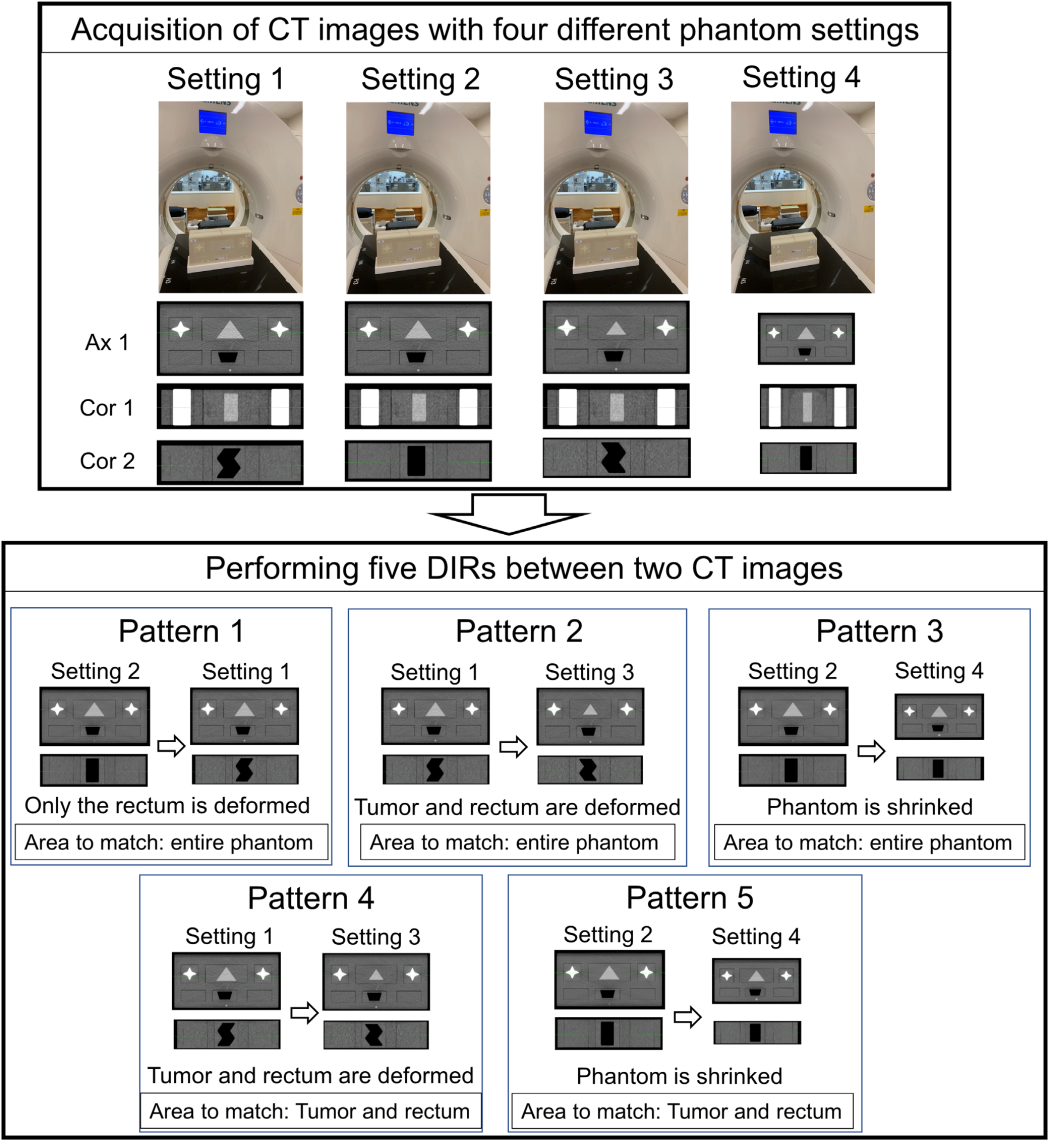
Overview of the combination of five deformable image registration (DIR) patterns. Four CT scans are acquired using different inserts and base phantoms. Then, DIR is performed using five DIR patterns between the two CT images.

We developed five DIR patterns using the combination of four phantom configurations (Fig. 3). All four phantom settings shared the following inserts: octagonal tough bone inserts (femoral heads) in slots 1 and 3 and blank inserts in slots 4 and 6. Only phantom setting 4 used the three‐quarter‐scaled base phantom. For phantom setting 1, the insert with a large trigonal acrylic object and the insert with an S‐shaped air cavity were inserted in slots 2 and 5, respectively. For phantom setting 2, the insert with a large trigonal acrylic and the insert with a trapezoidal air cavity were inserted in slots 2 and 5, respectively. For phantom setting 3, the insert with a small trigonal acrylic and the insert with an inverted S‐shaped air cavity were inserted in slots 2 and 5, respectively. For phantom setting 4, the three‐quarter‐scaled phantom was used and the insert with a large trigonal acrylic and the insert with a trapezoidal air cavity were inserted in slots 2 and 5, respectively.

Based on these four phantom settings, we determined five DIR patterns as follows.

- DIR pattern 1: phantom settings 1 (fixed image) and 2 (moving image). The area to match was the entire phantom.
- DIR pattern 2: phantom settings 3 (fixed image) and 1 (moving image). The area to match was the entire phantom.
- DIR pattern 3: phantom settings 4 (fixed image) and 2 (moving image). The area to match was the entire phantom.
- DIR pattern 4: phantom settings 3 (fixed image) and 1 (moving image). The area to match was focused only on tumor proxy and rectum proxy regions (i.e., body shape and femoral heads were ignored).
- DIR pattern 5: phantom settings 4 (fixed image) and 2 (moving image). The area to match was focused only on tumor proxy and rectum proxy regions.

DIR aims to determine the spatial transformation warping the moving image to match the fixed image as closely as possible.

In clinical practice, based on a request by radiation oncologists, we determined the area to match in the DIR software. For example, if radiation oncologists want to determine the cumulative dose distribution of initial and boost plans, we perform DIR to increase the DIR accuracy in the patient's body. If radiation oncologists want to determine the histogram parameters of the only cumulative dose volume of the rectum, we perform DIR to increase the DIR accuracy, particularly in the rectum. Based on these clinical purposes, we used two different clinical strategies in our credentialing workflow.

### 2.3 DIR instructions

The phantom with four phantom settings was scanned using the clinical computed tomography (CT) scan protocol for pelvic regions at each institution. The acquired CT images were transferred to the DIR software. Then, DIR was performed using five DIR patterns (Fig. 3). The institutions were asked to use the clinical DIR protocol for pelvic regions. If an institution did not have a DIR protocol for pelvic regions, the institutions were asked to use the DIR protocol that was most commonly used in the institution. Moreover, each institution could create contours of the structures of interest and use them for DIR, if needed. After completing DIR, the displacement vector fields (DVFs) of the five DIR patterns were exported into the DICOM format for MIM software (MIM Software Inc, Cleveland, OH, USA), bdf format for Velocity (Varian Medical Systems, Palo Alto, CA, USA), and mhd format for RayStation (RaySearch Laboratories, Stockholm, Sweden). Additionally, we asked each institution to fill out a form pertaining to the DIR procedure and parameter settings.

### 2.4 Evaluation

To create reference contours, which were used for contour‐based quantitative DIR evaluation, two experienced medical physicists and one radiation therapist created the contours of tumor, rectum, right femoral head, left femoral head, and body proxies in the CT images using the four phantom settings of the phantom by employing the MIM software (76 images: 4 phantom settings × 19 data). Deformed contours were created using DVFs submitted by each institution and three reference contours. Finally, the average Dice similarity coefficient (DSC) and Hausdorff distance (HD) for the tumor, rectum, right femoral head, left femoral head, and body proxies were calculated using the three reference and deformed contours in the fixed image for each pattern.^21^ DSC is a common measure of the spatial overlap between contours, and it is defined using the following formula:

(1) $$\text{DSC}=\frac{V_{d}\cap V_{s}}{\left(V_{d}\;+\;V_{s}\right)/2},$$

where *V_d_* is the volume of the contours deformed using DIR and *V_s_* is the volume of contours manually delineated on the reference CT image. HD is defined as the maximum closest distance between two volumes, where the closest distance is computed for each vertex of the two volumes. To reduce the uncertainty of the reference contour, we used the average DSC and HD using three reference contours.

To calculate the fiducial marker‐based target registration error (TRE), one medical physicist created a center of mass for 40 fiducial markers in CT images using the 4 settings of the phantom (i.e., the creation of a point‐to‐point correspondence between the moving and fixed images). TRE is defined as the three‐dimensional Euclidean distance between the fiducial marker coordinates on the fixed image and the corresponding fiducial marker coordinates on the deformed images. Further detailed methodology for calculation of TRE is described in the literature.^11^ To evaluate the HU difference in the CT images among institutions, the mean HU value was assessed using the right and left femoral head proxies.

The authors will not share research data.

## 3 RESULTS

End‐to‐end DIR credentialing was performed on 21 DIR software at 19 participating institutions in 2019 and 2020. Table 1 summarizes the detailed information of the CT scan and DIR software at all institutions. Overall, 17 domestic and 2 international institutions (21 datasets) participated in this study. Eight institutions used the MIM software, seven institutions used Velocity, and six institutions used RayStation. Two institutions participated using two types of DIR software.

**Table 1:** Detailed information of the CT scan and DIR software at each institution.

Institution	CT scanner	Scan mode	Slice thickness (mm)	Tube voltage (kVp)	X‐ray tube current (mA)	Convolution kernel	Dimensions	Voxel size (mm)	DIR software
1	Light Speed RT16 (GE Medical Systems)	Helical	2.5	120	203	Standard	512 × 512 × 39	0.98 × 0.98 × 2.5	MIM (6.6.9)
2–1	Light Speed RT16 (GE Medical Systems)	Axial	2.5	120	381	Standard	512 × 512 × 40	0.78 × 0.78 × 2.5	MIM (6.9.4)
2–2									RayStation (6.2.0.7)
3	Aquilion/LB (Canon)	Helical	2.0	120	300	FC13	512 × 512 × 71	1.07 × 1.07 × 2.0	MIM (6.9.4)
4–1	Aquilion ONE (Canon)	Helical	3.0	120	376	FC13	512 × 512 × 35	0.96 × 0.96 × 3.0	MIM (6.9.4)
4–2									RayStation (6.2.0.7)
5	Optima CT580 (GE Medical Systems)	Helical	1.25	120	381	Standard	512 × 512 × 81	0.98 × 0.98 × 1.25	MIM (6.4.9)
6	Aquilion/LB (Canon)	Helical	1.0	120	314	FC13	512 × 512 × 89	1.07 × 1.07 × 1.0	MIM (6.8.8)
7	Aquilion ONE (Canon)	Helical	2.0	120	267	FC03	512 × 512 × 61	0.98 × 0.98 × 2.0	MIM (6.9.2)
8	Aquilion/LB (Canon)	Helical	2.0	120	83	FC13	512 × 512 × 51	1.07 × 1.07 × 2.0	MIM (6.9.4)
9	Brilliance Big Bore (Philips)	Helical	3.0	140	132	B	512 × 512 × 42	1.17 × 1.17 × 3.0	Velocity (4.1)
10	Optima CT580 (GE Medical Systems)	Helical	2.5	120	647	Standard	512 × 512 × 41	1.27 × 1.27 × 2.5	Velocity (3.2.1)
11	SOMATOM Definition AS+ (Siemens)	Helical	2.0	120	125	B30s	512 × 512 × 51	0.98 × 0.98 × 2.0	Velocity (3.2.1)
12	Aquilion CXL (Canon)	Helical	2.0	120	169	FC13	512 × 512 × 71	0.96 × 0.96 × 2.0	Velocity (3.2.1)
13	Aquilion/LB (Canon)	Helical	2.0	120	50	FC13	512 × 512 × 51	1.07 × 1.07 × 2.0	Velocity (3.2.1)
14	Aquilion/LB (Canon)	Helical	2.0	120	392	FC13	512 × 512 × 51	0.78 × 0.78 × 2.0	Velocity (4.0)
15	SOMATOM Sensation Open (Siemens)	Helical	2.0	120	61	B30s	512 × 512 × 52	0.98 × 0.98 × 2.0	Velocity (3.2.1)
16	Aquilion PRIME (Canon)	Helical	2.0	120	130	FC13	512 × 512 × 56	0.98 × 0.98 × 2.0	RayStation (6.2.0.7)
17	Optima CT580 (GE Medical Systems)	Axial	2.5	120	336	Standard	512 × 512 × 40	1.17 × 1.17 × 2.5	RayStation (6.2.0.7)
18	SOMATOM Definition AS (Siemens)	Helical	2.0	120	115	B30f	512 × 512 × 51	0.98 × 0.98 × 2.0	RayStation (6.2.0.7)
19	Revolution HD (GE Medical Systems)	Helical	2.5	140	375	B	512 × 512 × 101	0.98 × 0.98 × 2.0	RayStation (9.0.0.113)

Table 2 presents a summary of DIR algorithms used for all datasets. For MIM, the participating institutions used one of the following three DIR algorithms: intensity‐based (M1), intensity‐based Reg Refine (M2), and hybrid DIR (M3). Reg Refine is the user‐guided deformable tool that provides user guidance pertaining to DIR using multiple rigid fusions with locked points.^22^ For velocity, the participating institutions used one of the following three DIR algorithms: deformable multipass (V1), extended deformable multipass (V2), and structure‐guided DIR (V3). For RayStation, all participating institutions used hybrid DIR without specific organ structures (R1) and hybrid DIR with specific organ structures (R2).

**Table 2:** Summary of DIR algorithms.

Institutions	Pattern 1	Pattern 2	Pattern 3	Pattern 4	Pattern 5
1	M2	M2	M2	M2	M2
2–1	M1	M1	M1	M1	M1
2–2	R2	R2	R2	R2	R2
3	M3	M3	M3	M3	M3
4–1	M1	M1	M1	M1	M1
4–2	R1	R1	R1	R2	R2
5	M1	M1	M1	M1	M1
6	M1	M1	M1	M1	M1
7	M2	M2	M2	M2	M2
8	M3	M3	M3	M3	M3
9	V1	V1	V1	V3	V3
10	V2	V2	V2	V2	V2
11	V2	V2	V2	V2	V2
12	V1	V1	V1	V3	V3
13	V2	V2	V2	V2	V2
14	V1	V1	V1	V1	V1
15	V3	V3	V3	V3	V3
16	R2	R2	R2	R2	R2
17	R2	R2	R2	R2	R2
18	R1	R1	R2	R2	R2
19	R1	R1	R2	R2	R2

Note

M1, MIM/intensity‐based DIR; M2, MIM/intensity‐based DIR with Reg Refine; M3, MIM/hybrid DIR; R1, RayStation/hybrid DIR without specific organ structures; R2, RayStation/hybrid DIR with specific organ structures; V1, Velocity/intensity‐based DIR (deformable multipass); V2, Velocity/intensity‐based DIR (extended deformable multi‐pass); V3 = Velocity/structure‐guided DIR.

**Fig. 4:**
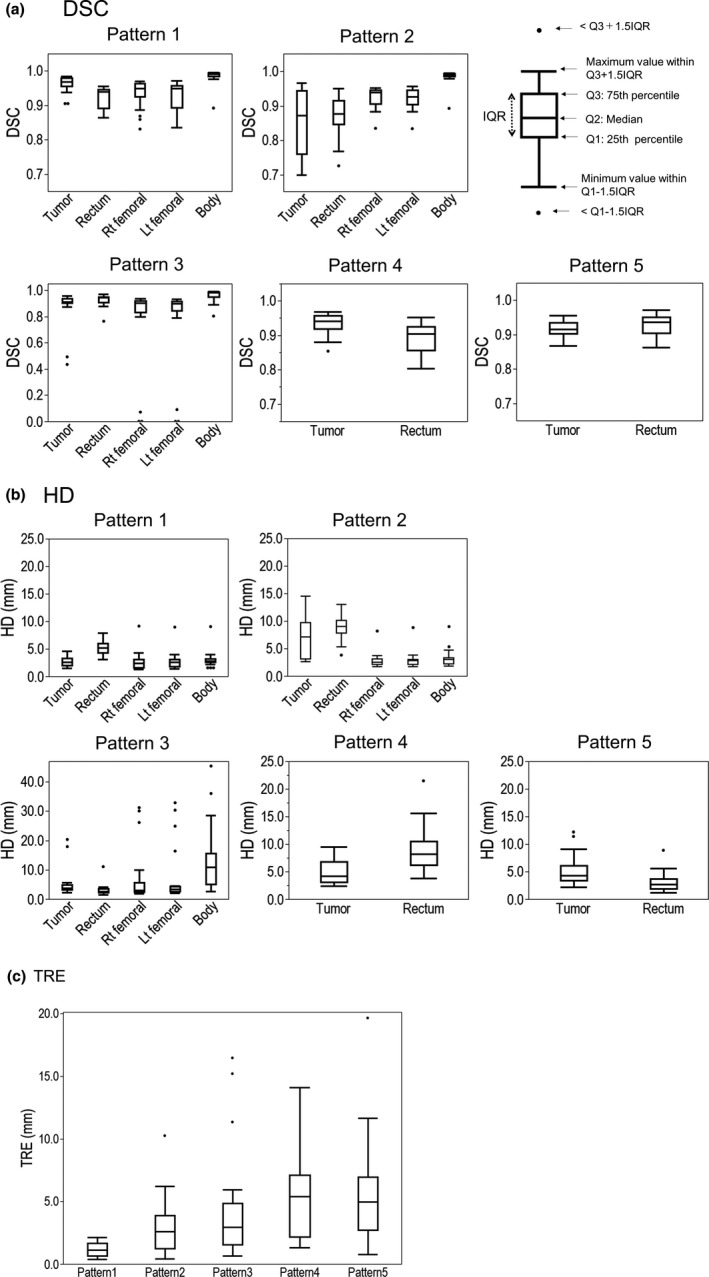
Box plots of (a) Dice similarity coefficient (DSC), (b) Hausdorff distance (HD), and (c) target registration error (TRE) in all DIR patterns.

**Fig. 5:**
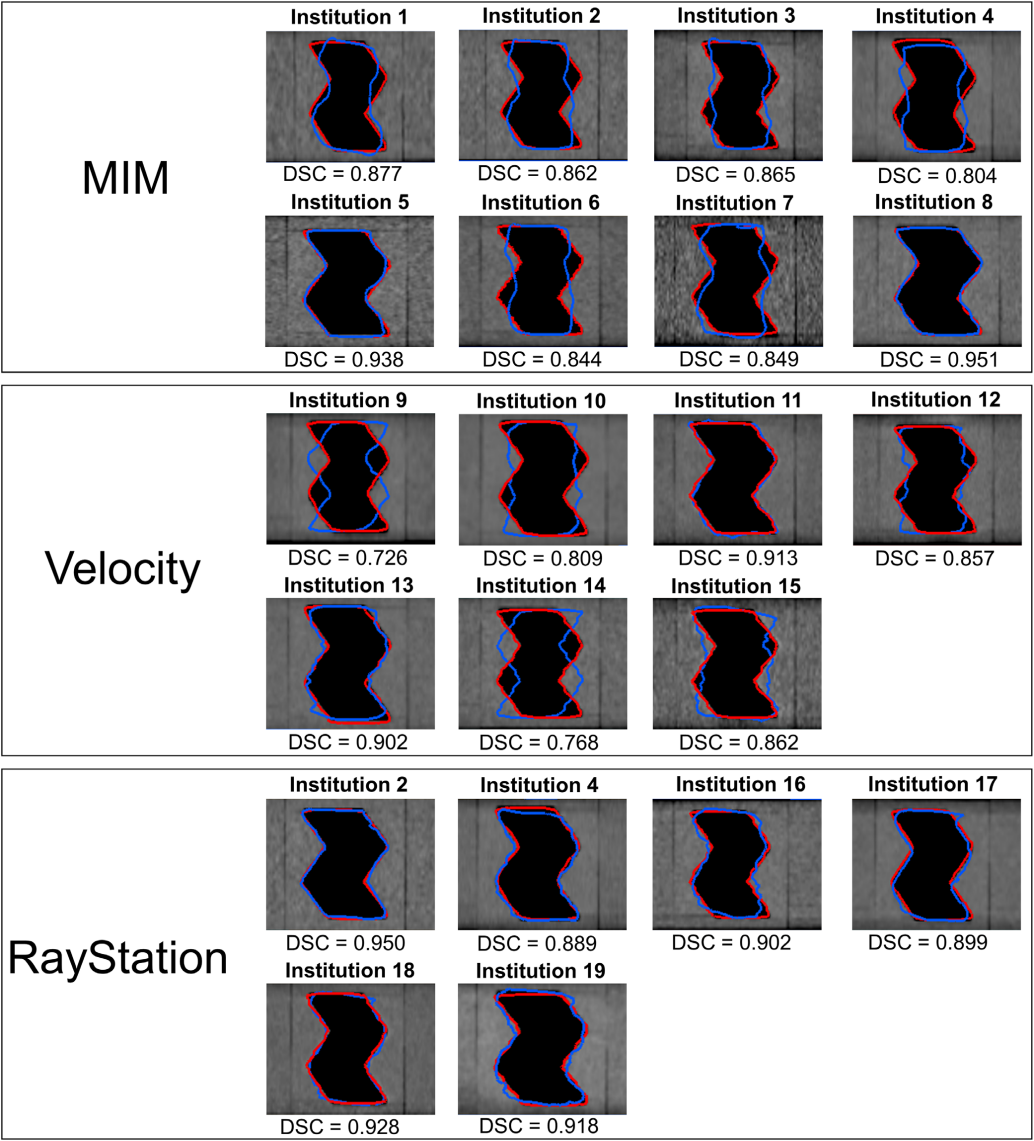
Fixed images (coronal images) with deformed and reference contours of the rectum proxy in deformable image registration (DIR) pattern 2. Red and blue lines indicate reference and deformed contours, respectively.

A summary of registration errors for each institution is shown in Fig. 4 and supporting information Table S1. The mean and one standard deviation (SD) values (range) of DSC in all patterns were 0.909 ± 0.088 (0.434–0.984) and 0.909 ± 0.048 (0.726–0.972) for tumor and rectum proxies, respectively. Moreover, 61.9% of data (13/21) had more than a DSC of 0.8 in both tumor and rectum proxies. A mean DSC of less than 0.8 was observed for DIR patterns 2 and 3 in the tumor proxy and DIR patterns 2 and 4 in the rectum proxy. The SDs of DSC in the rectum proxy were the highest in DIR pattern 2. The deformed and reference contours of the rectum proxy in the fixed image in DIR pattern 2 are shown in Fig. 5. Large variations were observed by visual inspection. Furthermore, the mean and one SD values of DSC in DIR patterns 1 and 2 (the bone was unchanged) were 0.929 ± 0.036 and 0.925 ± 0.035 for the right and left femoral head proxies, respectively. Moreover, the mean and one SD values of DSC in DIR pattern 3 (the bone was changed) were 0.771 ± 0.032 and 0.771 ± 0.031 for the right and left femoral head proxies, respectively.

The DIR accuracy was evaluated for DIR software types. For the DSC of the tumor proxy, the mean and one SD values in all DIR patterns were 0.940 ± 0.028 (MIM), 0.887 ± 0.108 (Velocity), and 0.893 ± 0.104 (RayStation). For the DSC of the rectum proxy, the mean and one SD values in all DIR patterns were 0.913 ± 0.047 (MIM), 0.886 ± 0.055 (Velocity), and 0.931 ± 0.029 (RayStation).

Moreover, the mean and one SD (range) values of HD for all the data in all DIR patterns were 5.02 ± 3.32 (1.53–20.35) and 5.79 ± 3.47 (1.22–21.48) (mm) for tumor and rectum proxies, respectively. Overall, the HD result was similar to the DSC result. For the HD of the tumor proxy, the mean and one SD values in all DIR patterns were 3.69 ± 1.46 (MIM), 6.27 ± 3.60 (Velocity), and 5.31 ± 4.15 (RayStation) (mm). For the HD of the rectum proxy, the mean and one SD values in all DIR patterns were 5.40 ± 3.25 (MIM), 7.10 ± 4.09 (Velocity), and 4.77 ± 2.45 (RayStation) (mm).

Additionally, the mean and one SD values (range) of TRE for all data were 1.15 ± 0.55 (0.38–2.13) (mm) (pattern 1), 2.98 ± 2.33 (0.41–10.26) (mm) (pattern 2), 4.35 ± 4.51 (0.66–16.47) (mm) (pattern 3), 5.19 ± 3.29 (1.33–14.09) (mm) (pattern 4), and 5.52 ± 4.20 (0.76–19.66) (mm) (pattern 5). These results show that DIR patterns 4 and 5 exhibited higher values than the other patterns.

The mean and one SD values (range) of HU were 754.3 ± 38.5 HU (670.7–803.3) and 766.4 ± 40.1 HU (687.1–815.0) for the right and left femoral head proxies, respectively.

## 4 DISCUSSION

The proposed physical DIR phantom was developed to ensure that end‐to‐end tests can be easily performed in various clinical settings that include tumor, rectum, and body changes. We tested the feasibility of DIR credentialing using the proposed phantom at multiple domestic and international institutions. The results provide feedback to the institutions that performed poorly, indicating where they may improve; these institutions were allowed to resubmit their results to demonstrate that they can meet the standards required for the trial.

The mean DSC values for all data were 0.434–0.984 and 0.726–0.972 in the tumor and rectum proxies. Regarding the DIR performance of each DIR software with different DIR algorithms, for the most severe pattern (i.e., pattern 2), mean DSCs in all regions (tumor, rectum, right femoral head, left femoral head, and body) were 0.913 ± 0.056 (M1, *n* = 4), 0.937 ± 0.045 (M2, *n* = 2), 0.949 ± 0.036 (M3, *n* = 2) for MIM; 0.837 ± 0.102 (V1, *n* = 3), 0.902 ± 0.062 (V2, *n* = 3), 0.878 ± 0.111 (V3, *n* = 1) for Velocity; and 0.904 ± 0.077 (R1, *n* = 3) and 0.933 ± 0.069 (R2, *n* = 3) for RayStation. Similar results were observed in other patterns. This finding suggests that advanced DIR techniques, which used the contour information or locked points (i.e., M2, M3, and R2), could greatly improve the DIR accuracy compared with intensity‐based DIR. Previous studies have shown that hybrid‐based DIR could improve the DIR accuracy in pelvic regions.^23^, ^24^ Kadoya et al. used the lung inhale and exhale images to evaluate three commercial DIR software.^11^ Loi et al. used synthetic images (head and neck, thorax, and pelvis sites) generated by specific DVF to test six commercial DIR software.^25^ These studies showed that the DIR performance strongly depended on the DIR software and procedure. Our result is consistent with their results. The variation among institutions using the same DIR software was mainly attributed to differences in the software version, rigid registration strategy, DIR algorithm, focusing area for DIR, and DIR parameter settings (e.g., grid size). Moreover, the participating institutions in all previous studies used the provided datasets; however, the participating institutions in our study used CT images acquired in each institution. For example, institutions 11 and 13 used the same DIR software with the same DIR version and procedures. However, a slight difference in the DIR accuracy was observed between two data (e.g., rectum proxy DSC, pattern 2: 0.913 vs. 0.902). Similar differences were observed between institutions 17 and 18. These results indicate that the CT scan protocol (i.e., slice thickness and convolution kernel) changes the DIR accuracy.

Regarding the DIR credentialing method, we used five DIR patterns. DIR patterns 2 and 3 showed a wider range of DSC and HD than other DIR patterns. DIR pattern 2 changed the tumor proxy and rectum proxy shapes and focused on the entire phantom. Further, DIR pattern 3 changed all proxy shapes and focused on the entire phantom, suggesting that these two patterns (patterns 2 and 3) were the severe condition for DIR. Thus, the differences in the DIR procedure among institutions may be sensitively reflected in the DIR accuracy.

Regarding the fiducial maker‐based TRE, DIR patterns 4 and 5 exhibited higher values than the other patterns. Because DIR patterns 4 and 5 focused only on the tumor and rectum proxies, participating institutions excessively attempted to increase the DIR accuracy in the tumor and rectum proxies (ignoring the DIR accuracy in other parts of the body proxy). More institutions used the contour information as the DIR guide (i.e., M3, V3, and R2 algorithms) in patterns 4 and 5 than in other patterns to increase the DIR accuracy in only the tumor proxy and rectum proxy regions. Consequently, unrealistic deformation may occur outside the tumor proxy region, where the fiducial markers were placed, resulting in higher TRE in DIR patterns 4 and 5 than in other DIR patterns. Based on these results, the workflow that used different DIR patterns with different matching areas can be useful for determining the DIR protocol (technical skills for DIR) in each institution, although the workflow of credentialing may depend on how DIR is used in clinical trials.

An important aspect of the DIR credentialing program is the establishment of acceptability criteria. AAPM TG 132 suggested a DSC value of 0.8 as the acceptance criteria (acceptance criteria for HD was not described). In three patterns (i.e., DIR patterns 1, 2, and 3), which evaluated the DIR accuracy in the entire phantom, 61.9% of the data (13/21) passed the criteria in the tumor proxy and rectum proxy regions. In the other two patterns (i.e., DIR patterns 4 and 5), which evaluated the DIR accuracy in the only tumor proxy and rectum proxy regions, 100% of the data passed the criteria. Because the physical phantom exhibits a simpler structure than the actual patient, the DIR accuracy tends to be high. In the future, we plan to determine the specific criteria for the proposed phantom by analyzing the relationship between quantitative (e.g., visual inspection using a scale of one to five) and qualitative results (e.g., DSC).

The limitations of this study are as follows. First, the proposed phantom was designed to assess the comprehensive DIR accuracy at each institution. Herein, we created a simple representation of the pelvic region by inserting an octagonal tough bone proxy, trigonal PMMA, and various air cavities. By rearranging the inserts or including inserts that are hollow inside, we can simulate the simple thoracic region in the proposed phantom. Although this phantom may be used for DIR evaluation in other clinical sites, we only evaluated the DIR accuracy in one clinical site (i.e., pelvis). Second, a limitation of this geometric phantom is that the deformations are simple and linear (reflections and linear expansions). For example, the rectum proxy goes from a consistent zigzag shape to a straight line. Real pelvic deformations for the prostate were influenced by the bladder and rectum motions (e.g., filling and gas passage). These aspects could be addressed using an anthropomorphic phantom and or more complicated inserts in the geometry of this phantom. Third, the three‐quarter‐scaled phantom used in pattern 3 was created by uniformly scaling down the entire phantom, including the custom inserts. Although this phantom may present an unlikely clinical scenario, we used this design to increase the sensitivity for determining the technical skills for DIR. Fourth, our results may include residual errors caused by the variability in the placement of the inserts between two phantom settings. When we use the proposed phantom for the DIR credentialing of clinical trials, we must carefully pay attention to the custom insert position. Fifth, we used inserts comprising geometric shapes with distinct patterns (i.e., many corners), which may be influenced by CT scanning parameters (e.g., CT resolution and slice thickness); however, this influence may not be prominent in clinical situations. Sixth, only three types of DIR software were used in this study.

## 5 CONCLUSIONS

We developed a physical phantom for the DIR credentialing of a clinical trial. We revealed a wide range of DIR evaluation parameters among the participating institutions. Thus, the proposed method shows considerable potential as an evaluation tool for DIR credentialing and QA.
